# Overview of lithium's use: a nationwide survey

**DOI:** 10.1186/s40345-020-00215-z

**Published:** 2021-03-09

**Authors:** Xabier Pérez de Mendiola, Diego Hidalgo-Mazzei, Eduard Vieta, Ana González-Pinto

**Affiliations:** 1 Bioaraba, Research Group on Severe Mental Illness; Osakidetza, Araba University Hospital, Psychiatry Service; Faculty of Medicine, Department of Neurosciences, University of the Basque Country UPV / EHU, Vitoria-Gasteiz, Spain; 2 Hospital Clinic, Institute of Neuroscience, University of Barcelona, IDIBAPS, Centre for Biomedical Research Network on Mental Health (CIBERSAM), Barcelona, Spain

**Keywords:** Lithium, Bipolar disorder, Survey, Psychiatrists’ attitude, Adverse effects, First episode, Adolescence onset, Dosing schedule

## Abstract

**Background:**

Lithium is considered the gold standard treatment for bipolar disorder (BD). Current clinical guidelines and scientific evidence support its use as a first-line treatment in BD. However, over the last two decades, there has been a downward tendency in lithium's use in several developed countries. Based on a nationwide survey, this study's objective is to analyze in a large sample of psychiatrists relevant issues of the use of lithium salts in BD.

**Methods:**

Data were collected through an anonymous survey sent by email among 500 psychiatrists who belong to a National Society of Psychiatry (Spanish Society of Biological Psychiatry). The survey is a self-administered questionnaire consisting of 21 items on the most key aspects of lithium's use (indication, dosage, monitoring, and information for patients).

**Results:**

212 psychiatrists completed the survey. 70% of psychiatrists prescribe lithium to more than 50% of patients diagnosed with BD. Adverse effects are the main reason not to use lithium salts. Over 75% of the participants consider lithium salts the treatment of choice for the maintenance phase of BD, both in women and men. Most of the participants (> 50%) start lithium after the first affective episode, use conservative plasma concentrations (0.6–0.8 mmol/L), and generally prescribe it twice a day. 57% of psychiatrists who treat patients under 18 do not use lithium in this population. About 70% of the survey respondents use official protocols to inform and monitor patients on lithium treatment.

**Conclusions:**

From the results of the present study, it can be concluded that the use of lithium in Spain is in line with the recommendations of the main international clinical guidelines and current scientific literature. The first reason not to prescribe lithium in our country is the perception of its adverse effects and not the aspects related to its practical use or its effectiveness. Considering that BD is a chronic disease with a typical onset in adolescence, the low rate of prescription of lithium salts in patients under 18 must be thoroughly studied.

## Introduction

Even though more than 70 years have passed since the Australian psychiatrist John Cade reported the antimanic efficacy of lithium carbonate (Cade 1949), the main current clinical guidelines still consider it a first-choice treatment for Bipolar Disorder (BD) (Fountoulakis et al. 2017; Yatham et al. 2018). It has proved useful not only in acute manic episodes (Yildiz et al. 2014) but also in depressive (Baldessarini et al. 2020; Malhi et al. 2017) and mixed episodes (Sani and Fiorillo 2019). Nevertheless, lithium is noted for its outstanding efficacy in the maintenance or prophylactic treatment of BD (Jauhar and Young 2019; Severus et al. 2018). Bearing in mind the frequent chronic, recurrent and disabling nature of BD (a disease that affects more than 1% of the world population) (Vieta et al., 2018), a long-term treatment which allows preventing relapses or recurrences is vital. In this regard, lithium continues to be the gold standard treatment supported by extensive scientific evidence (Carvalho et al., 2020). Both in controlled clinical trials and observational studies, lithium has shown its efficacy and superiority in the prophylaxis of any type of affective episode (Berk et al. 2017; González-Pinto et al. 2018; Kessing et al. 2018; Lähteenvuo et al. 2018; Miura et al. 2014; Severus et al. 2014).

In addition to its mood-stabilizing properties, lithium has a distinctive, independent, and proven anti-suicide action (Barjasteh-Askari et al. 2020; González-Pinto et al. 2006; Smith and Cipriani 2017; Song et al., 2017). This is a relevant quality in BD since up to 15% of patients diagnosed with BD die by suicide (Gordovez and McMahon 2020). In fact, a systematic review and meta-analysis showed that treatment with lithium among people with mood disorders could reduce the risk of death and suicide up to 60% compared to placebo (Cipriani et al. 2013). Remarkable neuroprotective and antiviral properties have also been attributed to lithium (Post 2018; Rybakowski 2018; Murru et al. 2020; Van Gestel et al. 2019). It slows brain aging (Van Gestel et al. 2019) and reduces the risk of dementia by almost 50% in patients with BD (Velosa et al. 2020). It could also attenuate the cognitive and functional decline in patients (without BD) with mild cognitive impairment (Forlenza et al. 2019). Moreover, the use of lithium has recently been proposed as a potential treatment for CoViD-19 (Murru et al. 2020).

Despite the undeniable evidence in favor of its application in BD, a descendent tendency in the use of lithium has been noticed in the US (Rhee et al. 2020) and in numerous European countries (Bohlken et al. 2020; Karanti et al., 2016; Kessing et al., 2016; Lyall et al., 2019). In several of them, it has changed from being the most prescribed drug to the least one, even behind the controversial antidepressants. (Kessing et al., 2016; Lyall et al., 2019). The emergence of new effective drugs for BD, such as second-generation antipsychotics and certain antiepileptics, has overturned the prescription pattern of BD (Malhi et al., 2020; Anmella et al., 2020). The absence of pharmaceutical marketing, the toxic perception of its adverse effects, the slow onset of action and the need for venipuncture monitoring are some of the possible causes of this declining trend (Gitlin, 2016a; Rybakowski, 2018). Neither the main clinical guidelines nor current scientific literature supports the idea of replacing lithium with other drugs. In fact, over the last two decades, the decrease in lithium's use is not widespread, and in certain countries, lithium's prescription rate has remained high. In some countries, more than 50% of bipolar patients are treated with lithium, while in the US, for example, only 17% (Kessing 2019; Parabiaghi et al. 2015; Renes et al. 2018; Rhee et al. 2020) (Table 1).

**Table 1:** Changes in the lithium prescription rate in several European countries over the last two decades

Study	Country	Period	Data source	Results
Bohlken et al. 2020	Germany	2009–2018	Neuropsychiatric private practices' records	The percentage of patients with bipolar disorder receiving lithium declined from 31,4% (2009) to 26,2% (2018)
Rhee et al. 2020	United States	1997–2000 vs. 2013–2016	Outpatient physician reports of patient visits	The percentage of patients with bipolar disorder receiving lithium declined from 30,4% (1997–2000) to 17,6% (2013–2016)
Lyall et al. 2019	Scotland	2009–2016	Records of outpatient clinic attendance, general/acute hospital admissions and psychiatric hospital admissions	The percentage of patients with bipolar disorder receiving lithium declined from 26% (2009) to 22% (2016)
Renes et al. 2018	Netherlands	2009–2014	Outpatient psychiatrists’ and patients’ surveys	Lithium was used by 70% of patients with bipolar disorder or schizoaffective disorder, bipolar type
Karanti et al. 2016	Sweden	2007–2013	Records of private and public psychiatric outpatient health care units	The percentage of patients with bipolar disorder receiving lithium declined from 51% (2007) to 41% (2013)
Kessing et al. 2016	Denmark	2000–2011	Records of all Danish patients with a first-ever contact with mental healthcare	The one-year prescription rate of lithium in bipolar patients decreasedfrom 41% (2000) to 34% (2011)
Parabiaghi et al. 2015	Italy	2000–2010	A population-based database of dispensing records	The prevalence of lithium treatment grew by 38% duringthe observation period
Hayes et al. 2011	England	1995–2009	Records of primary care patients	The prescriptionrate for lithium increased from 22.5% (1995) to 29.3% (2009)
Castells et al. 2006	Spain	1985–2003	Pharmacy sales data of medicinal products	Lithium daily dose per 1000 inhabitants per day (DID) increased from 0.21 (1985) to 0.79 (2003)

In this context, an anonymous survey was carried out among psychiatrists. The primary objective of this study was to evaluate the use and current perception of lithium's treatment on the most relevant aspects of the drug: indication, dosage, monitoring, and information for patients. The results presented below could be used to elaborate new consensus or national protocols, which will promote and optimize the use of lithium in our country.

## Methods

### Study design and sample

Between 11 May and 11 July 2020, an anonymous online survey was conducted on the use of lithium, thanks to the support of the Spanish Society of Biological Psychiatry (SEPB). All the members of the SEPB (500 psychiatrists) received an email in which they were informed about the purpose of the study. They were invited to participate in it by completing the questionnaire attached to the same message. The collaboration was voluntary, and responses were recorded, eliminating the identity of the participants. Two reminder emails were sent during the mentioned period to maximize the response rate. The survey was created and performed with Google Forms, and Microsoft Office Excel 2019 was used for the analysis and representation of the data. The study was approved by the Clinical Research Ethics Committee of the Araba University Hospital (Spain).

### Questionnaire

The survey was designed to obtain a general perspective on lithium's use from a sample of Spanish psychiatrists. In order to encourage participation, a brief self-administered questionnaire (21 items) with multiple-choice questions (Additional file 1: Appendix S1) was elaborated. The first 5 questions are related to demography: age, sex, and origin of the respondents. The following 3 points address the prescription rate of lithium in BD and the main reasons not to prescribe it. The next 5 requests explore its status compared to the rest of the drugs and the stage in the course of the illness when lithium is usually introduced. Subsequently, there are 2 specific questions about two practical and relevant aspects of lithium's use: plasma concentrations and dose distribution. The next 4 refer to the use of lithium in special clinical circumstances (minors, elderly and psychiatric comorbidity). The final issues are about the availability of official documents or protocols to monitor and inform patients undergoing lithium treatment.

### Analysis

Data are analyzed using descriptive statistics. The results are represented by graphs based on percentages and absolute numbers using Microsoft Office Excel 2019.

## Results

### Demographics

A total of 212 responses are obtained from almost all Spain regions (Additional file 1: Appendix S2. Figure S1). The majority of respondents (86%) come from Catalonia (28%), the Community of Madrid (27%), Basque Country (15%), Andalusia (8%), and Valencian Community (8%). The distribution between sexes and age groups is practically homogeneous as it is the percentage of professionals who work at hospitals (psychiatric or general) and outpatient settings (Table 2).

**Table 2:** Demographic profile of the participants

Profile of psychiatrists	n (%)
**Sex**	
Man	100 (47%)
Woman	112 (53%)
**Age range**	
25–35	54 (26%)
36–45	57 (27%)
46–55	42 (20%)
56–65	39 (18%)
> 65	20 (9%)
**Work center**	
General hospital	88 (41%)
Psychiatric hospital	23 (11%)
Outpatient consultation	101 (48%)
**Total**	212 (100%)

### Lithium's prescription and reasons not to use it

Only 3% of respondents never prescribe lithium salts. 70% of participants prescribe lithium to more than 50% of patients diagnosed with BD and 20% to more than 75% of them (Additional file 1: Appendix 2. Figure S2).

**Fig. 1:**
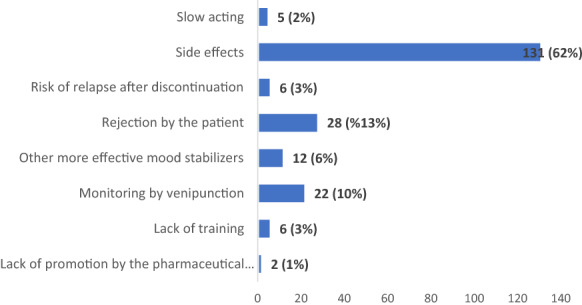
Main reasons not to prescribe lithium

Almost 62% of psychiatrists affirm that the main reason not to prescribe lithium is its side effects. A significantly lower percentage of specialists state that the leading cause not to prescribe it is the rejection by the patient (13%) and the need for monitoring by venipuncture (10%) (Fig. 1).

Over 75% of the participants choose lithium as the first option in the maintenance treatment of BD, both in women and men (Additional file 1: Appendix S2. Figure S3). As a second option, antipsychotics (34%) and valproate (31%) stand out in women. For men, valproate (64%) is clearly the second preferred option.

Finally, more than 80% of respondents usually initiate treatment with lithium salts after the first affective episode. Most of them prescribe it after the first manic episode. More than 25% of psychiatrists, when there is a family history of BD, tend to start lithium treatment after the first depressive episode (Additional file 1: Appendix S2. Table S1).

### Serum levels and dose distribution

**Fig. 2:**
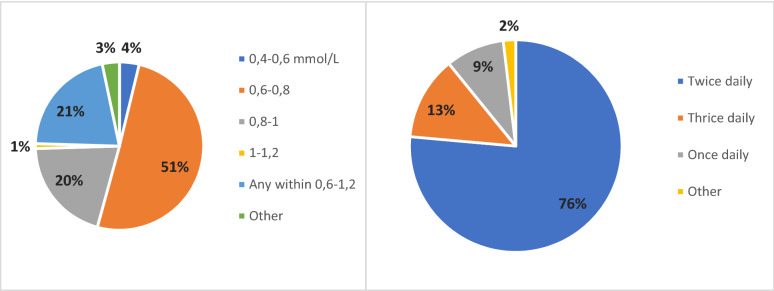
The preferred serum level and dose distribution of lithium

50% of the participants use serum lithium levels between 0.6 and 0.8 mmol/L for the maintenance phase of BD. 21% consider adequate any concentration within the therapeutic range established between 0.6 and 1.2 mmol/L. Another 20% utilizes higher lithium serum levels between 0.8 and 1 mmol/L to prevent relapse or recurrence in BD (Fig. 2).

Regarding the distribution of the lithium dose, more than 75% of psychiatrists prescribe it in 2 daily doses. 13% in 3 daily doses and only 9% in a single daily dose (Fig. 2).

### Specific populations

60% of respondents (128) do not deal with children and adolescents. Among the psychiatrists who care for underage BD patients, most (57%) do not use lithium salts in this population (Additional file 1: Appendix S2. Table S2). By contrast, the majority of survey respondents do use lithium in older age BD (Additional file 1: Appendix S2. Table S2).

Over 80% of specialists do not have any trouble prescribing lithium to patients with a comorbid Substance Use or a Personality Disorder (Additional file 1: Appendix S2. Table S2).

### Information for patients and monitoring protocols

One-third of psychiatrists who prescribe lithium do not have documentation for patients at their workplace. Furthermore, a quarter of respondents do not follow a formal protocol for monitoring lithium and its adverse effects (Additional file 1: Appendix S2. Figure S4).

## Discussion

This is the first study referring to the use of lithium that analyzes psychiatrists' current general perspective. In 2018, an interesting international survey was published focused exclusively on lithium monitoring (Nederlof et al. 2018). In order to understand the international framework of lithium's underuse, it is essential to know the specialists' point of view (Malhi et al. 2020).

According to the National survey results on the use of lithium, the vast majority of Spanish psychiatrists in this sample, which includes professionals from all the autonomous communities, comply with the recommendations of the latest clinical guidelines and the scientific literature. 70% of the survey respondents prescribed lithium to more than 50% of patients diagnosed with BD and 20% to over 75%. In this line, more than 75% of national psychiatrists who participated in this study chose lithium as their first-choice treatment in the maintenance phase of BD both in men and women.

The apparently high lithium's prescription by Spanish psychiatrists agrees with the recently observed results in the Netherlands, where 70% of patients diagnosed with BD or Schizoaffective Disorder were treated with lithium (Renes et al. 2018). Nevertheless, as it is mentioned above, this percentage is significantly higher than in other neighboring countries (Kessing 2019). In Sweden, the prescription rate for lithium in BD is 55%, in Denmark 41.7%, in Germany 26.2%, and in Scotland 22% (Bohlken et al. 2020; Karanti et al. 2016; Kessing et al. 2016; Lyall et al. 2019) (Table 1).

In the current study, we asked participants about the reasons not to prescribe lithium in the long-term treatment of BD. Most agree that lithium's adverse effects are the main barrier to its use. Only 6% of the participants believe that lithium's non-prescription is due to the availability of other more effective mood stabilizers. This fact shows the conviction that participating psychiatrists have in the efficacy of lithium above other effective drugs. Practical aspects related to lithium's use, such as the need for monitoring or the slow onset of action, are not considered obstacles for the drug's prescription. As suggested for clozapine (Bachmann et al. 2017; Verdoux et al. 2018), the high use of lithium in Spain could be the consequence of a local "culture" that favors the use of the drug. This could be because of the transmission of personal experience from expert therapists to beginners, institutional support that facilitates the adequate infrastructure for patients' follow-up on lithium treatment (lithium clinics and national registries, for example), and the promotion of its use by scientific societies. Knowing exactly why lithium is not prescribed in other countries would help to understand better its international underuse.

Despite the fact that most of the professionals believe that lithium's side effects are the main limiting factor for its prescription, more than 75% consider it the first-choice treatment for the maintenance therapy of BD for both women and men. In recent years, it has been confirmed that the most severe adverse effects of lithium, that is, kidney dysfunction and teratogenic risk, were overestimated in the former reports (Fornaro et al. 2020; Nielsen et al. 2017). Additionally, the risk of suffering from both complications can be minimized by using a minimum effective dose and close monitoring of plasma levels (Tondo et al. 2019). To avoid making the survey extensive and more challenging to answer, we decided not to ask about the short- and long-term adverse effects of lithium in the current study, although we did consider it. Now that we know this result, it would be interesting to conduct a new survey in the future, focusing on the adverse effects of lithium and its management.

Therapeutic alternatives to lithium are not exempted from significant risks. Valproate has a high teratogenic risk, discouraging its use in women of childbearing age (European Medicines Agency 2018; Anmella et al. 2019). That is why it is striking the relatively high percentage of psychiatrists (31%) who choose valproate as a second option for women suffering from BD. On the other hand, antipsychotics are associated with a more significant weight gain and a worse metabolic profile than lithium (Hayes et al. 2016; Jauhar and Young 2019). Besides, they can cause extrapyramidal and sexual symptoms, too (Huhn et al. 2019). Although it is clear that there is a reasonable concern about its negative effects, these data would explain the high percentage of psychiatrists who choose lithium as the first option for the long-term treatment of BD in both sexes. Moreover, lithium has potential long-term benefits related to neurogenesis that are being studied (Berk et al. 2017; Forlenza et al., 2019; Sun et al., 2019; Zanni et al., 2019; Ciftci et al. 2020). Emphasizing the benefits of lithium without forgetting its adverse effects (Gitlin 2016b; Tondo et al. 2017) could help improve the outcome of patients with BD.

The most used serum lithium levels for the maintenance treatment of BD (0.6–0.8 mmol/L) are also in line with current scientific advice (Nolen et al. 2019). This is a crucial issue because the use of conservative plasma levels could prevent lithium intoxication, renal, and central nervous system adverse effects (Nielsen et al. 2018; Schoot et al. 2019). Another practice that could minimize the risk of renal impairment is the schedule of a single lithium daily dose (Schoot et al. 2019). However, this regimen is the least used among the respondents who prefer to distribute lithium dosage in 2 or 3 daily doses.

Another point that differs from the latest scientific publications is the little use of lithium among children and adolescents with BD. Most psychiatrists who take care of minors (57%) do not use lithium when the literature suggests that it is an effective and safe treatment in this population (Amerio et al. 2018; Hafeman et al. 2019). Considering that the typical onset of the disease is in adolescence, and due to the dividing line between child and adult care in Spain, it can be challenging to supply lithium since the first episode.

Finally, it must be stated that the availability of official documentation on lithium for patients (67%) and protocols for its monitoring (72%) is similar to the mentioned international study (Nederlof et al. 2018). The publication of expert consensuses, such as the one which has recently been prepared by the SEPB (González-Pinto et al. 2021), is an initiative that can improve these figures.

This study has several strengths and limitations. As underlined before, this is the first research that evaluates the general perspective on lithium's use among a large group of psychiatrists. The fact that 86% of the responses come from 5 autonomous communities limits the generalization of the results to the whole country. Nonetheless, they are 5 of the most populated autonomous communities, representing 64% of the Spanish population. So, this is a somewhat expected finding. Furthermore, these 5 regions attract specialists trained in other territories because of their greater job offer. The number (212) and the rate (42.5%) of responses are higher or similar to other surveys addressed to professionals (Campos et al. 2020; Daod et al. 2019; Nederlof et al. 2018). The social desirability and selection bias could have overestimated the use of lithium in our country. The survey was distributed only among members of a single national society of Psychiatry, not considering the perspective of psychiatrists who are not members of the SEPB. As part of SEPB, members might be exposed to a more continuous update and training on guidelines due to the continuous education and training opportunities offered within the society. Research participants could have chosen the most socially desirable or acceptable responses rather than responses that reflected their true thoughts or practices. However, the anonymous nature of the survey could have favored obtaining honest and real answers.

Finally, the present study does not analyze national prescription registers. Thus, it would be interesting to complement the current essay with a pharmaco-epidemiological analysis, such as those carried out in other countries that have already been pointed out (Table 1). Nevertheless, it should be noted that the results obtained in this study are congruent with the pharmaco-epidemiological research performed in other countries (Parabiaghi et al. 2015; Renes et al. 2018) and with the only one published in Spain (Castells et al. 2006). In a forthcoming paper, it would also be interesting to explore lithium's use in the acute phases (mania, depression, and mixed states). Its efficacy and role are different from the maintenance phase and vary across the distinct acute phases (Baldessarini et al. 2020; Malhi et al. 2017). Moreover, most recent guidelines state that clinicians should consider the maintenance phase when selecting acute phase treatments (Yatham et al. 2018).

## Conclusions

This survey results suggest that, at least in Spain, the use of lithium is consistent with the latest clinical guidelines. Based on these results, except for children and adolescents, one cannot speak of an underuse of lithium in Spain. The use of valproate as one of the main alternatives to lithium in women is one of the few matters that differ from the recommendations of the main scientific societies. The main barrier to prescribe lithium is its side effect profile. Behind this apparent high use of lithium could be a local culture that favors the dissemination of scientific and practical information about the drug.

### Supplementary Information

**Additional file 1: Appendix S1.** The questionnaire. **Appendix S2:** Complementary figures and tables.

## Data Availability

The datasets used and/or analyzed during the current study are available from the corresponding author on reasonable request.

## Abbreviation

BD: Bipolar disorder
