# Correction: Overview of lithium’s use: a nationwide survey

**DOI:** 10.1186/s40345-024-00343-w

**Published:** 2024-07-25

**Authors:** Xabier Pérez de Mendiola, Diego Hidalgo-Mazzei, Eduard Vieta, Ana González-Pinto

**Affiliations:** 1 Faculty of Medicine, Department of Neurosciences, Bioaraba, Research Group on Severe Mental Illness; Osakidetza, Araba University Hospital, Psychiatry Service; University of the Basque Country UPV / EHU, Vitoria-Gasteiz, Spain; 2 Hospital Clinic, Institute of Neuroscience, University of Barcelona, IDIBAPS, Centre for Biomedical Research Network on Mental Health (CIBERSAM), Barcelona, Spain

**Correction to: Int J Bipolar Disord (2021) 9:10**

10.1186/s40345-020-00215-z.

In this article [1], there was an error in the Funding number. The correct Funding should be as below.

Funding

This work was supported by Carlos III Health Research Institute [Grant Number PI18/01055] (co-financed by the European Regional Development Fund (FEDER/ERDF)/European Social Fund ‘Investing in your future’); Networking Center for Biomedical Research in Mental Health (CIBERSAM), the Basque Government [Grant number, 2017111104] and the University of the Basque Country [Grant Number 321212ELBY]. The psychiatric research department in Araba University Hospital is supported by the Stanley Research Foundation [Grant number 03-RC-003].
