# Supplementary material for: Overview of lithium's use: a nationwide survey
Source: Int J Bipolar Disord. 2021 Mar 9;9:10. doi: 10.1186/s40345-020-00215-z (PMC7941362; doi:10.1186/s40345-020-00215-z)
Supplement: Supplementary file 1 — Additional file 1: Appendix S1. The questionnaire. Appendix S2: Complementary figures and tables. [file 40345_2020_215_MOESM1_ESM.docx]

**Supplementary Appendix S1.** The questionnaire

**NATIONAL SURVEY ON LITHIUM'S USE**

* Please check only one answer.

1. Please indicate your gender
   - Man
   - Woman
2. Please indicate your age group
   - 25-35 years
   - 36-45
   - 46-55
   - 56-65
   - > 65
3. Please indicate in which province you work.
4. Please indicate in which municipality you work.
5. Please indicate what type of health center you work at.
   - Outpatient setting
   - General hospital
   - Psychiatric hospital
   - Other:
6. Do you prescribe lithium salts for patients with Bipolar Disorder (BD)?
   - Yes
   - No
7. If so, about what percentage of patients with BD do you prescribe lithium to?
   - 0-25%
   - 25-50%
   - 50-75%
   - 75-100%
8. What do you think is the main reason not to prescribe lithium in BD?
   - Availability of other more effective mood stabilizers
   - Adverse effects of lithium
   - Slow onset of action
   - Need for monitoring by venipuncture
   - Patient's refusal of lithium treatment
   - High risk of relapse after discontinuation
   - Other:
9. What treatment do you use as the 1st option for the maintenance treatment of BD in women?
   - Antidepressants
   - Antipsychotics
   - Lithium
   - Lamotrigine
   - Valproate
   - Other antiepileptics
   - Other:
10. What treatment do you use as the 2nd option for the maintenance treatment of BD in women?
    - Antidepressants
    - Antipsychotics
    - Lithium
    - Lamotrigine
    - Valproate
    - Other antiepileptics
    - Other:
11. What treatment do you use as the 1st option for the maintenance treatment of BD in men?
    - Antidepressants
    - Antipsychotics
    - Lithium
    - Lamotrigine
    - Valproate
    - Other antiepileptics
    - Other:
12. What treatment do you use as the 2nd option for the maintenance treatment of BD in men?
    - Antidepressants
    - Antipsychotics
    - Lithium
    - Lamotrigine
    - Valproate
    - Other antiepileptics
    - Other:
13. At what point in the course of the illness do you usually prescribe lithium for the maintenance treatment of BD?
    - After the 1st manic episode
    - After the 1st depressive episode when there is a family history of BD
    - In the two previous situations
    - During the first 5 years of the illness
    - During the first 5-10 years of the illness
    - After more than 10 years of the illness
    - I do not prescribe lithium
    - Other:
14. What range of serum lithium levels do you use for the maintenance phase of BD?
    - 0,4-0,6 mmol/L
    - 0,6-0,8 mmol/L
    - 0,8-1 mmol/L
    - 1-1,2 mmol/L
    - Any one within the 0.6-1.2 mmol/L range
    - I do not prescribe lithium
    - Other:
15. How many times a day do you usually prescribe lithium?
    - Once a day in the morning
    - Once a day in the evening
    - Twice a day
    - Three times a day
    - Other:
16. Do you prescribe lithium to minors with BD?
    - Yes
    - No
    - I do not treat minors
17. Do you prescribe lithium in older adults with BD?
    - Yes
    - No
    - I do not treat older adults
18. Do you prescribe lithium to patients with bipolar disorder and a comorbid substance use disorder?
    - Yes
    - No
    - I do not treat patients with a substance use disorder
19. Do you prescribe lithium to patients with BD and a comorbid personality disorder?
    - Yes
    - No
    - I do not treat patients with a personality disorder
20. Do you have documentation for patients on lithium treatment at your workplace?
    - Yes
    - No
    - I do not prescribe lithium
21. Do you follow any official protocol for monitoring lithium treatment and its adverse effects?
    - Yes
    - No
    - I do not prescribe lithium

**Supplementary Appendix S2.** Complementary figures and tables

**Figures:**

**Figure S1**. The number of responses in each Autonomous Community. Only regions with data are displayed.


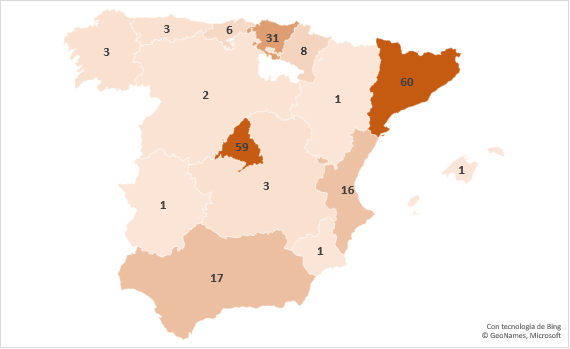


**Figure S2**. Percentage of patients with BD who are treated with lithium.

**Figure S3**. The preferred treatments for maintenance therapy in BD.

**Figure S4.** Availability of official documentation.

**Tables:**

**Table S1**. The time when lithium is started in BD.

| After the 1st manic episode | 111 (52,5%) |
| --- | --- |
| After the 1st depressive episode with a family history of BD | 8 (4%) |
| In the two previous situations | 51 (24%) |
| During the first 5 years of the disease | 28 (13%) |
| Between the first 5-10 years of the disease | 6 (3%) |
| 10 years after the onset of the disease | 1 (0,5%) |
| I do not prescribe lithium | 7 (3%) |
| Total | 212 (100%) |

**Table S2**. The use of lithium in specific populations with BD.

|  | **N** | **%** |
| --- | --- | --- |
| **Children and adolescents** |  | |
| YES | **36** | **43%** |
| NO | **48** | **57%** |
| **Older adults** |  | |
| YES | **145** | **76%** |
| NO | **45** | **24%** |
| **Substance Use Disorder** |  | |
| YES | **148** | **81%** |
| NO | **35** | **19%** |
| **Personality disorders** |  | |
| YES | **172** | **86%** |
| NO | **29** | **14%** |
